# The effect of comorbidities for the prognosis of community-acquired pneumonia: an epidemiologic study using a hospital surveillance in Japan

**DOI:** 10.1186/s13104-019-4848-1

**Published:** 2019-12-19

**Authors:** Mai Thi Ngoc Nguyen, Nobuyuki Saito, Yukiko Wagatsuma

**Affiliations:** 1 Department of Clinical Trials and Clinical Epidemiology, Graduate School of Comprehensive Human Sciences, University of Tsukuba, 1-1-1 Tennodai, Tsukuba, Ibaraki 305-8575 Japan; 2 The Shock and Trauma Center, Nippon Medical School Chiba Hokusoh Hospital, 1715 Kamagari, Inzai, Chiba 270-1694 Japan; 3 Department of Clinical Trials and Clinical Epidemiology, Faculty of Medicine, University of Tsukuba, 1-1-1 Tennodai, Tsukuba, Ibaraki 305-8575 Japan

**Keywords:** Community-acquired pneumonia, Mortality, Comorbidity, Charlson Comorbidity Index

## Abstract

**Objective:**

Pneumonia is a common but serious illness that continues to present significant morbidity and mortality. Although the effect of severity at admission on outcome has been well reported, the role of comorbidity is still not widely understood. The Charlson Comorbidity Index measures comorbidity with a well-established history of predicting long-term outcome but its utility in pneumonia prognosis is still limited. Here, we use the Charlson Comorbidity Index and hospital surveillance data to investigate associations between comorbidities and in-hospital mortality due to community-acquired pneumonia.

**Results:**

Among the 535 eligible adult patients (69.0% male, median [IQR] age, 79 [70–84] years), 100 (18.7%) acquired severe to extremely severe pneumonia. The median [IQR] CCI was 1 [1–3]. Malignancy (129 of 535, 24.1%), chronic pulmonary diseases (113 of 535, 21.1%) and congestive heart failure (103 of 535, 19.3%) were frequent. Higher Charlson Comorbidity Index scores were associated with higher risk of in-hospital mortality (OR 1.28; 95% CI 1.07–1.53). These results support the inclusion of comorbid burden in predicting community-acquired pneumonia outcome.

## Introduction

Despite the universal availability of antibiotics and adherence to recommended treatment guidelines, pneumonia continues to present significant morbidity and mortality. Pneumonia and influenza combined ranked eighth among the most common causes of death in the United States and pneumonia alone accounted for more than 60,000 deaths in 2005 [1]. In an international systematic review, the overall mortality was 13.7%, ranging from 5.1% in hospitalized and ambulatory patients to 36.5% for the intensive care unit patients [2].

Based on the surveillance data, the estimated annual number of adult community-acquired pneumonia (CAP) was 1.9 million for entire Japan, 69% of which were in the elderly [3]. In-hospital death rates sharply increase with age, making CAP the third leading cause of death among older adults in Japan. Influenza vaccine and pneumococcal vaccine are both recommended for the elderly by the national guideline in Japan. However, the coverages of these vaccinations are insufficient. Recent studies reported the estimated coverage of 57% for influenza vaccination [4] and 34% for pneumococcal vaccine among the elderly in Japan [5].

Comorbidity is essential in risk prediction and risk adjustment modeling. Previous works have suggested using a diagnosis-based index, such as the Charlson Comorbidity Index (CCI), in studies where the outcome of interest is mortality [6]. The CCI contains 19 conditions, each of which is assigned a weight of 1, 2, 3 or 6 based on adjusted hazard ratios for each comorbid condition derived from Cox’s proportional hazards model [7]. These weighted scores are then summed into a total score. Numerous adaptations of the CCI have been developed for use in administrative databases [8–10], all of whom independently developed coding algorithms using the International Classification of Diseases, Ninth Revision (ICD-9). In 2005, Quan et al. [11] identified the ICD-10 codes corresponding to the Deyo ICD-9 adaption and updated their index in 2011, creating the latest version of CCI [12].

This updated CCI index has been proven competent at predicting mortality in various disease subgroups, including cancer, renal disease, stroke, or in patients undergoing surgical procedures. However, in spite of various efforts to utilize the index to predict CAP prognosis from 1 to up to 7 years after hospital discharge, studies have tended to focus on long-term rather than short-term outcomes. Accordingly, reports on the influence of CCI on CAP in-hospital mortality and its application to clinical settings are scarce. This study thus aimed to investigate the association between comorbidities, as assessed by CCI, and in-hospital mortality due to community-acquired pneumonia.

## Main text

This was a retrospective cohort study conducted at the 600-bed Nippon Medical School Chiba Hokusoh Hospital that specializes in cancer and emergency care. Information was extracted from the Diagnosis Procedure Combination database (DPC) and discharge database. DPC is a nationwide administrative database for acute-care inpatients, which first emerged in 2003, and is linked to a lump-sum payment system. As of 2012, the DPC is estimated to cover approximately 53% of general hospital beds in Japan [13]. In this study, the DPC database provided information on the severity of the pneumonia in the form of an A-DROP score. Remaining data were obtained from the discharge database, including identification codes, main and subcategorized diagnoses, patient demographics, past medical history, summarized clinical courses, admission and discharge status and discharge medication. Diagnoses were coded using ICD-10.

### Study subjects

The present study included data of patients admitted between August 1, 2011 to June 30, 2017. All consecutive emergency room admissions ≥ 18 years of age with the diagnosis of pneumonia (ICD-10 codes: J18.0, J18.1, J18.2, J18.8, J18.9) were considered for inclusion. To validate community-acquired pneumonia (CAP) diagnoses, all cases were reviewed and those with clinical symptoms of pneumonia (e.g., dyspnea, cough, fever, sputum production, chest pain) that demonstrated infiltration on chest radiography within 48 h of admission were selected. Patients were excluded if they were later defined as having health care-associated pneumonia or hospital-acquired pneumonia, if they presented with cardiopulmonary arrest on admission, consented to a do-not-resuscitate order or had incomplete data. Patients referred from or transferred to another hospital were also excluded.

### Measures

The primary outcome was all-cause, in-hospital mortality. Independent variables of interest were age, sex, pneumonia severity (A-DROP score), the primary predictor of CCI and its comorbid conditions component. Between one and six points were awarded for each condition and summed, with higher scores correlating to higher severity of comorbidity. Comorbid diseases were retrieved from past medical histories and, for each disease, the corresponding ICD-10 code was identified [14]. From this ICD code, the CCI was calculated by applying the coding algorithm and weight assignment [11, 12].

The severity of pneumonia on admission was evaluated using the A-DROP prognostic score, a modified version of the CURB-65 scoring system proposed by the Japanese Respiratory Society that is well-established and has a proven ability to predict mortality [15, 16]. Age, dehydration status, respiratory failure, orientation disturbance, and blood pressure were assessed before stratifying patients into four severity classes based on total score. Age, the length of stay (LOS) and CCI were recorded and analyzed as continuous variables.

### Data analysis

Due to potential bias concerns, analysis was performed twice: first, with all participants and then after excluding severe and extremely severe cases with no record of comorbidity (CCI = 0). Categorical variables are presented in absolute numbers and proportions while continuous variables are expressed as median (interquartile range). Univariate logistic regression analyses were first performed to evaluate each covariate’s individual effect on the outcome. All covariates of interest, together with their interaction term, were then entered into the multivariable logistic models and analyses were repeated after exclusion of severe to extremely severe patients with no comorbidity records. Model diagnostics were also performed which tested for the assumptions of linearity of logit (for variable age and CCI), multicollinearity, and influential cases. Odds ratios are reported with 95% confidence intervals and p values are 2-sided with a p < 0.05 cutoff for statistical significance. All analyses were performed with SPSS (version 23, IBM Inc., USA).

## Results

Data from 660 individuals who were admitted to the emergency department with a pneumonia diagnosis from August 2011 to June 2017 were extracted. Of the 125 excluded cases, 64 had other principal diagnoses (e.g., lung cancer, interstitial lung *diseases*), 33 were later classified as either hospital-acquired or health-care associated pneumonia, 5 had an incomplete record, 6 had cardiopulmonary arrest on admission, 4 consented to a do-not-resuscitate order, and 13 were either transferred from or transferred to another hospital. Thus, a total of 535 cases were left for analysis.

The median age (IQR) of patients was 79 (70–84) years, 369 of 535 (69.0%) were men, and all-cause, in-hospital mortality rate was 12% (Table 1). The cohort stayed for approximately 14 days in the hospital. In term of pneumonia severity, the majority was classified into the moderate group (338 individuals, 68.0%) whereas 100 patients (18.7%) had pneumonia at severe or extremely severe levels. The median CCI (IQR) was 1 (1–3) with roughly a quarter of the patients (131 individuals, 24.5%) scoring 2 points. The most common co-morbidities found in the CCI were malignancies (129 of 535, 24.1%), chronic pulmonary diseases (113 of 535, 21.1%) and congestive heart failure (103 of 535, 19.3%). In general, excluding cases with no record of comorbidities, there was a trend towards increasing mortality as the CCI score increased. The number rose from 6.8% in patients with CCI = 1 to 25.0% with CCI = 5.

**Table 1:** General characteristics of study subjects

Characteristics	N = 535
Age in years, median (IQR)	79 (70–84)
Sex, male, n (%)	369 (69.0)
Length of stay in days, median (IQR)	14 (9–27)
Charlson Comorbidity Index, median (IQR)	1 (1–3)
In-hospital mortality, % (n/N)	12.2 (64/535)

*IQR* interquartile range

Univariable logistic regression modeling showed that older patients who presented with more severe pneumonia at admission were at higher risk for in-hospital death as each 1-year increase in age heightened the risk by 4% (odds ratio [OR] 1.04; 95% confidence interval [CI] 1.01 to 1.07). There was a trend towards increasing mortality in patients with malignancy, congestive heart failure, renal disease, diabetes with complication and dementia. The correlation between pneumonia severity and the CCI was weak, i.e., Spearman’s correlation coefficient = 0.139, p = 0.002. The multivariable regression model showed there was a tendency toward increasing mortality as A-DROP score increased but only in the extremely severe patients showed a significant difference. Among mild pneumonia patients, the prognostic impacts of CCI were marginally significant after adjustments for age and sex as a 1-point rise in CCI increased odds of death by twofold (OR 2.09; 95% CI 1.03–4.25). This impact, however, was reduced significantly as pneumonia severity increased (Additional file 1: Table S1).

Considering a potential bias, we conducted the final analysis after excluding severe and extremely severe patients whose CCI was zero (60/353; 17.0%) and found that each per unit increase in CCI resulted in a 35% increase in odds of death (OR 1.35; 95% CI 1.16 to 1.58) (Table 2). Renal disease, followed by malignancy, showed the strongest association with mortality (OR 2.42; 95% CI 1.13 to 5.21 and OR 2.25; 95% CI 1.21 to 4.17). Age, sex, A-DROP categories, CCI and their interaction terms were added as predictors for multivariate analysis but no meaningful interactions were observed. The prognostic impacts of CCI were consistent across pneumonia severity categories and a 1-point increase in CCI increased odds of death by 28% (OR 1.28; 95% CI 1.07–1.53) (Table 3).

**Table 2:** Univariate analysis for in-hospital mortality

Variables	n (%)	OR (95% CI)	p-value
Age, years^a^	475 (88.8)	1.04 (1.01–1.07)	0.01
Sex			
Male	329 (61.5)	Reference	
Female	146 (27.3)	1.18 (0.63–2.24)	0.61
Pneumonia severity			
Mild A-DROP = 0	68 (12.7)	Reference	
Moderate A-DROP = 1–2	338 (63.2)	1.28 (0.43–3.80)	0.66
Severe A-DROP = 3	52 (9.7)	4.29 (1.28–14.39)	0.02
Extremely severe A-DROP = 4–5	17 (3.2)	11.20 (2.77–45.31)	< 0.01
Charlson Comorbidity Index (CCI)^b^	475 (88.7)	1.35 (1.16–1.58)	< 0.01
Charlson Comorbidity Index components^c^			
Any malignancy			
No	349 (73.5)	Reference	
Yes	126 (26.5)	2.25 (1.21–4.17)	0.01
Chronic pulmonary disease			
No	367 (77.3)	Reference	
Yes	108 (22.7)	1.04 (0.51–2.13)	0.91
Congestive heart failure			
No	375 (78.9)	Reference	
Yes	100 (21.1)	1.89 (0.98–3.65)	0.06
Renal disease			
No	422 (88.8)	Reference	
Yes	53 (11.2)	2.42 (1.13–5.21)	0.02
Diabetes with complication			
No	434 (91.4)	Reference	
Yes	41 (8.6)	2.03 (0.84–4.87)	0.11
Dementia			
No	435 (91.6)	Reference	
Yes	40 (8.4)	2.10 (0.87–5.04)	0.10
Rheumatologic disease			
No	459 (96.6)	Reference	
Yes	16 (3.4)	1.31 (0.29–5.97)	0.72
Mild liver disease			
No	460 (96.8)	Reference	
Yes	15 (3.2)	0.64 (0.08–5.00)	0.67

*OR* odds ratio, *CI* confidence interval

^a^OR for 1 year increasing in age

^b^OR for one point increasing in CCI

^c^Conditions which has less than 10 cases were not analyzed

**Table 3:** Multivariable logistic regression analysis for in-hospital mortality

Variables	No. of deaths/total number	B	OR (95% CI)	p-value
Age, years^a^	47/475	0.03	1.03 (0.99–1.07)	0.08
Sex				
Male	31/329		Reference	
Female	16/146	0.34	1.41 (0.71–2.83)	0.33
Pneumonia severity				
Mild A-DROP = 0	4/68		Reference	
Moderate A-DROP = 1–2	25/338	− 0.31	0.73 (0.22–2.41)	0.61
Severe A-DROP = 3	11/52	0.59	1.80 (0.47–6.96)	0.39
Extremely severe A-DROP = 4–5	7/17	1.60	4.92 (1.11–21.98)	0.04
Charlson Comorbidity Index (CCI)^b^	47/475	0.25	1.28 (1.07–1.53)	< 0.01

*B* beta coefficient, *OR* odds ratio, *CI* confidence interval

^a^OR for 1 year increasing in age

^b^OR for one point increasing in CCI

## Discussion

This study found that higher CCI scores were associated with increased risk for all-cause, in-hospital mortality and this association persisted after adjustment for potential confounders. This interesting result stems from the fact that, although the index has been proven reliable for the long-term prognosis of CAP, there has been little discussion on how it influences short-term outcomes. In their study of 108 patients, Franzen et al. [17] reported that pneumonia in-hospital mortality trended upward as CCI rose (HR 1.10; 95% CI 0.99 to 1.23). In another study conducted on 373 Japanese patients, Torres and colleagues [18] were unable to find any impact of CCI on the mortality of elderly patients with CAP but details regarding A-DROP scores and direction of the associations were not reported.

Notably high proportions of this population suffered from malignancy (24.1%), chronic pulmonary diseases (21.1%) and congestive heart failure (19.3%). Studies conducted in other Japanese teaching hospitals have reported that approximately 8.8% to 19.0% of patients had malignancy while 5.4% to 12.7% suffered from congestive heart failure [19–21]. Our result was not unexpected, as the Chiba Hokusoh Hospital serves as a Chiba Cancer Treatment Regional Base Hospital and therefore has a considerable number of patients undergoing cancer treatment. In addition, since the average age was 79 years old, this would have contributed to increased complications.

We surprisingly discovered that patients with severe conditions (A-DROP = 3–5) but no comorbidities had the highest mortality rate at 48.0%. Contrary to expectations, individuals in the most severe group who had higher CCI seemed to survive better while those who scored zero on the CCI tended to die. One possible explanation is that these severe cases might receive treatment priority due to physician triage and collecting the past medical history was only done once the emergency situation was resolved. Patients who died quickly were more likely to have their disease history recorded incompletely and, as a result, they would tend to score 0 on the CCI. We posit that if information had been more thoroughly collected in these patients, their CCI scores would have been higher and have truly reflected their comorbidities. Sensitivity analyses were carried out to minimize this classification bias and, although a notable improvement observed in the results has strengthened our hypothesis, further studies with large representative samples are required to confirm our observation.

## Limitations

This study was conducted using retrospective data of a rural tertiary teaching hospital in Japan. The inclusion criteria were limited only to patients with a principal diagnosis of pneumonia. Several factors associated with mortality were not considered. Furthermore, the study could not identify deaths outside of the hospital that may have been tied to the pneumonia diagnosis.

## Supplementary information

**Additional file 1: Table S1.** Multivariable logistic regression analysis for in-hospital mortality.

## Data Availability

The datasets used and/or analyzed during the study are available from the corresponding author on reasonable request.

## Abbreviations

CAP: community-acquired pneumonia
CCI: Charlson Comorbidity Index
CI: confidence interval
DPC: Diagnosis Procedure Combination database
ICD: International Classification of Diseases
ICU: intensive care unit
IQR: interquartile range
OR: odds ratio
