# Supplementary material for: The effect of comorbidities for the prognosis of community-acquired pneumonia: an epidemiologic study using a hospital surveillance in Japan
Source: BMC Res Notes. 2019 Dec 19;12:817. doi: 10.1186/s13104-019-4848-1 (PMC6923893; doi:10.1186/s13104-019-4848-1)
Supplement: Supplementary file 1 — Additional file 1: Table S1. Multivariable logistic regression analysis for in-hospital mortality. [file 13104_2019_4848_MOESM1_ESM.docx]

Table S1. Multivariable logistic regression analysis for in-hospital mortality^a^

| Variables | | No. of death/total number | B | OR (95% CI) | p-value |
| --- | --- | --- | --- | --- | --- |
| Age, years^b^ | | 62/506 | 0.03 | 1.03 (0.99 – 1.07) | 0.10 |
| Sex | |  |  |  |  |
|  | Male | 32/352 |  | reference |  |
|  | Female | 20/154 | 0.35 | 1.42 (0.71-2.83) | 0.32 |
| Pneumonia severity | |  |  |  |  |
|  | Mild  A-DROP=0 | 4/68 |  | reference |  |
|  | Moderate  A-DROP=1-2 | 25/338 | 0.95 | 2.59 (0.30-22.45) | 0.34 |
|  | Severe  A-DROP=3 | 15/68 | 1.92 | 6.84 (0.67-69.86) | 0.11 |
|  | Extremely severe  A-DROP=4-5 | 18/32 | 4.63 | 101.97 (9.85-1055.18) | <0.01 |
| Charlson Comorbidity Index^c^ | | 62/506 | 0.74 | 2.09 (1.03-4.25) | 0.05 |
| CCI × pneumonia severity | |  |  |  |  |
| CCI × mild | |  |  | reference |  |
| CCI × moderate | |  | -0.57 | 0.57 (0.27-1.19) | 0.13 |
| CCI × severe | |  | -0.51 | 0.60 (0.27-1.32) | 0.20 |
| CCI × extremely severe | |  | -1.12 | 0.33 (0.14-0.76) | <0.01 |

Abbreviations: B, beta coefficient; OR, odds ratio; CI, confidence interval; ^a^Excluded cases with missing value on A-DROP scores; ^b^OR for one year increasing in age; ^c^OR for one point increasing in CCI
